# Targeting TF-AKT/ERK-EGFR Pathway Suppresses the Growth of Hepatocellular Carcinoma

**DOI:** 10.3389/fonc.2019.00150

**Published:** 2019-03-15

**Authors:** Shan-Zhou Huang, Meng-Ning Wei, Jia-Rong Huang, Zi-Jian Zhang, Wen-Ji Zhang, Qi-Wei Jiang, Yang Yang, Huan-Yu Wang, Hui-Lin Jin, Kun Wang, Zi-Hao Xing, Meng-Ling Yuan, Yao Li, Xiao-Shun He, Zhi Shi, Qi Zhou

**Affiliations:** ^1^Department of Hepatic Surgery, The First Affiliated Hospital, Sun Yat-sen University, Guangzhou, China; ^2^Organ Transplant Center, The First Affiliated Hospital, Sun Yat-sen University, Guangzhou, China; ^3^Guangdong Provincial Key Laboratory of Bioengineering Medicine, Department of Cell Biology and Institute of Biomedicine, National Engineering Research Center of Genetic Medicine, College of Life Science and Technology, Jinan University, Guangzhou, China; ^4^Department of Hepatobiliary Surgery, The Seventh Affiliated Hospital of Sun Yat-sen University, Shenzhen, China; ^5^Department of Thyroid and Breast Surgery, Nanshan District People's Hospital, Shenzhen, China; ^6^Department of General Surgery, Hui Ya Hospital of The First Affiliated Hospital, Sun Yat-sen University, Huizhou, China

**Keywords:** hepatocellular carcinoma, tissue factor, epidermal growth factor receptor, AKT/ERK, tumor growth

## Abstract

Tissue factor (TF) is a transmembrane glycoprotein to initiate blood coagulation and frequently overexpressed in a variety of tumors. Our previous study has showed that the expression of TF is upregulated and correlated with prognosis in hepatocellular carcinoma (HCC). However, the role and molecular mechanism of TF in the growth of HCC are still unclear. *In vitro* and *in vivo* functional experiments were performed to determine the effect of TF on the growth of HCC cells. A panel of biochemical assays was used to elucidate the underlying mechanisms. TF could promote the growth of HCC *in vitro* and *in vivo* by activating both ERK and AKT signaling pathways. TF induced EGFR upregualtion, and inhibition of EGFR suppressed TF-mediated HCC growth. In addition, TF protein expression was correlated with EGFR in HCC tissues. TF promotes HCC growth by upregulation of EGFR, and TF as well as EGFR may be potential therapeutic targets of HCC.

## Introduction

Hepatocellular carcinoma (HCC) is the fifth most lethal cancers worldwide, while China accounted for more than half of all cases and deaths in 2012 (1). More than 400,000 people die from liver cancer and over 450,000 new cases are diagnosed in China each year (2). Though the treatments for HCC have been greatly advanced in recent years, the outcome of HCC is still unoptimistic. Postoperative recurrence, the main reason for poor survival of HCC patients, mainly owes to the tendency of the invasion and metastasis of HCC cells (3, 4). Therefore, understanding the mechanism of HCC tumorigenesis and progression is critical to improve the clinical outcome of HCC patients.

Tissue factor (TF, also known as platelet tissue factor, factor III, thromboplastin, or CD142, encoded by the F3 gene) is a 47 kD transmembrane glycoprotein that contains 263 amino acid residues totally including a 219 amino acid extracellular region, a 23 amino acid hydrophobic transmembrane region, and a 21 amino acids C-terminal intracellular tail (5). Originally, TF is found on the surface of intravascular cells, such as platelets, leukocytes, and endothelial cells and functions as the principal initiator of the extrinsic coagulation cascade by binding with circulating factor VII or VIIα (FVII/VIIα) (6). Recently, TF is frequently overexpressed in a variety of tumors, including breast cancer, colorectal carcinoma, gastric cancer, non-small cell lung, and pancreatic ductal carcinoma, etc. (7). We and other groups have reported that the expression of TF is upregulated and correlated with prognosis in HCC (8–10). In the current study, we investigate the role and molecular mechanism of TF in the growth of HCC cells.

## Materials and Methods

### Patients and Tissue Specimens

A total 144 HCC tissues were obtained from patients who underwent curative resection between Jan 2008 and Dec 2010 at the First Affiliated Hospital, Sun Yat-sen University. None of the patients received neoadjuvant radiotherapy or chemotherapy before surgery. Signed informed consents were obtained from all patients. The study was approved by the ethics committee of the First Affiliated Hospital, Sun Yat-sen University.

### Cell Culture and Reagents

The human HCC cell lines HepG2, BEL-7402, SK-HEP1, SMMC-7721, and normal hepatic cell line LO2 were from China Center for Type Culture Collection and cultured in Dulbecco's modified Eagle's medium (DMEM) supplemented with 10% fetal bovine serum (FBS), penicillin (100 U/ml) and streptomycin (100 ng/ml) in a humidified incubator at 37°C with 5% CO_2_ atmosphere. U0126, LY294002, and Gefitinib were from ApexBio. Anti-TF (ab17375) and Anti-Ki-67 (2724-1) were from Abcam. Anti-pAKT (4060), Anti-AKT (4691), Anti-pERK (4370), and Anti-ERK (4695) antibodies were from Cell Signaling Technologies. Anti-EGFR (SC-03) and Anti-c-Myc (SC-40) antibodies were from Santa Cruz Biotechnology. Anti-β-actin (LK9001T) and Anti-GAPDH (LK9002T) antibodies were from Tianjin Sungene Biotech.

### Plasmid Construction and Lentivirus Production

The human TF cDNA was cloned into pLVX-AcGFP1-N1 lentiviral vector, and shRNA targeting human TF mRNA (5′-GCGCUUCAGGCACUACAAA-3′) was cloned into pLKO.1 lentiviral vector. Lentivirus was packaged in HEK293T cells and collected from the medium supernatant. Stable cell lines were established by infecting lentivirus into cells, followed by puromycin selection (11, 12).

### siRNA Transfection

The EGFR siRNA (sense sequences: 5′- CUGACUCCGUCCAGUAUUGAU−3′) and negative control siRNA were synthesized by Guangzhou Ribobio. Each siRNA solution was mixed gently with the respective volume of the X-tremeGENE siRNA Transfection Reagent and allowed to form transfection mixture for 20 min. Cells were cultured in 6-well plate with DMEM until 50% of confluence and added with the transfection mixture for 24 h before the next experiment (13, 14).

### Western Blot

Cells were harvested and washed twice with cold PBS, then resuspended and lysed in RIPA buffer (1% NP-40, 0.5% sodium deoxycholate, 0.1% SDS, 10 ng/ml PMSF, 0.03% aprotinin, 1 μM sodium orthovanadate) at 4°C for 30 min. Lysates were centrifuged for 10 min at 14,000 × g and supernatants were stored at −80°C as whole cell extracts. Proteins were separated on 12% SDS-PAGE gels and transferred to polyvinylidene difluoride membranes. Membranes were blocked with 5% BSA and incubated with the indicated primary antibodies. Corresponding horseradish peroxidase-conjugated secondary antibodies were used against each primary antibody. Signals were detected using the ChemiDoc XRS chemiluminescent gel imaging system (Bio-RAD) (15, 16).

### MTT Assay

Cells were seeded into a 96-well plate at a density of 0.5–1 × 10^4^ cells/well and treated with various concentrations of agents. After 3 days, 3-(4, 5-dimethylthiazolyl-2)-2, 5-diphenyltetrazolium bromide (MTT) was added to each well at a final concentration of 0.5 mg/ml. After incubation for 4 h, the medium and MTT solution were removed from each well, and formazan crystals were dissolved in 100 μl of DMSO. Absorbance was measured at 570 nm by Multiscan Spectrum (Thermofisher) (17, 18).

### Sphere Formation Assay

Cells were trypsinized, suspended in medium containing 0.3% agar and 10% FBS and seeded at a density of 5 × 10^2^ cells/well in a 12-well plate. The agar–cell mixture was plated onto a bottom layer with 0.5% agar. Then treated cells were incubated in a humidified incubator and fresh medium was added every 3 days. Two weeks later, colonies were analyzed microscopically (19, 20).

### Nude Mice Xenograft Tumor Assay

The female Balb/c nude mice with 5 weeks old and 16–18 g weight were obtained from the Shanghai SLAC Laboratory Animal Co and maintained with sterilized food and water. For xenograft tumor assay, 4 × 10^6^ cells in 100 μl of DMEM were injected subcutaneously under the shoulder of six mice per group. The mice were anesthetized after experiment, and tumors or lungs were removed, weighed, and sectioned. All experimental procedures were approved by the Institutional Animal Care and Use Committee of Jinan University (21, 22).

### Immunohistochemistry Assay

Immunohistochemistry (IHC) assay was performed with a microwave-enhanced avidin-biotin staining method. Formalin-fixed, paraffin embedded human HCC tissue array and subcutaneous tumors in mice were stained with antibodies, respectively, using a microwave-enhanced avidin-biotin staining method. To quantify the protein expression, the following formula was used: IHC score = percentage of positive cells × intensity score. The intensity was scored as follows: 0, negative (no staining); 1, weak (light yellow); 2, moderate (yellow brown); and 3, intense (brown) (23, 24).

### Statistical Analysis

Statistical analyses were performed using SPSS 19.0 for Windows (SPSS) and Graph-Pad Prism 6. Data were expressed as the mean ± standard deviation (SD) from at least three independent experiments. Quantitative data between two groups were compared using the Student's *t*-test. Categorical data were analyzed by the χ^2^ test or Fisher exact test. Correlations between different protein expressions level were determined using Spearman's rank analysis. The *p* < 0.05 was considered as statistical significance. ^*^p < 0.05; ^**^p < 0.01; NS: no statistical significance.

## Results

### Knockdown of TF Inhibits the Growth of HCC

**Figure 1:**
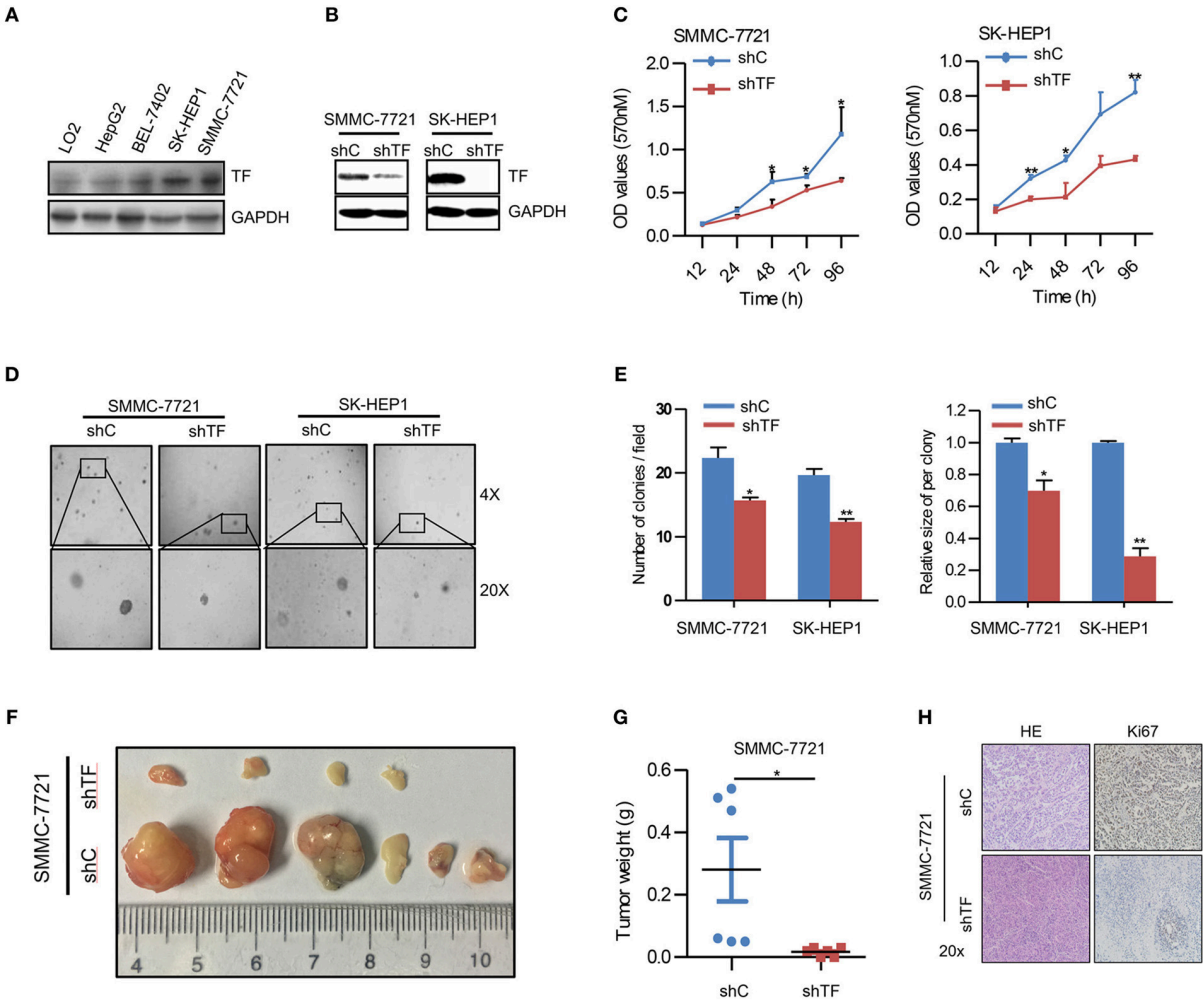
Knockdown of TF inhibits the growth of HCC. **(A,B)** Western blot analysis of the protein expressions in the indicated cells. **(C)** Cell growth of the indicated cells as determined with MTT assay. **(D)** Representative images and **(E)** quantification of the indicated cells sphere as determined with sphere formation assay. **(F)** The indicated subcutaneous tumors and **(G)** tumor weight of nude mice were shown. **(H)** Representative images of H&E and Ki-67 staining in the indicated tumor sections as determined with IHC assay. Error bars, mean ± SD. *p < 0.05 and **p < 0.01 [two-tailed Student's *t*-test **(C,E,G)**].

To explore the potential biological function of TF in HCC, we first examined the protein expression of TF in human HCC cell lines including HepG2, BEL-7402, SK-HEP1, SMMC-7721, and normal hepatic cell line LO2. Notably, all HCC cell lines displayed higher protein levels of TF than normal hepatic cell line, and SK-HEP1 and SMMC-7721 cells showed the highest protein levels of TF in all cells (Figure 1A). To further investigate the role of TF in HCC malignancy, we generated the cells with shRNA-mediated stable knockdown of endogenous TF in both SK-HEP1 and SMMC-7721 cells (Figure 1B). Knockdown of TF decreased the cell amounts, sphere numbers and sizes in both SK-HEP1 and SMMC-7721 cells as detected by MTT and sphere formation assays (Figures 1C–E). Additionally, the data of subcutaneous tumor models in nude mice showed that TF knockdown inhibited the growth of SMMC-7721 xenografts by decreasing the volumes and weights of tumors as well as the numbers of Ki67^+^ proliferating cells (Figures 1F–H).

### Overexpression of TF Promotes the Growth of HCC

**Figure 2:**
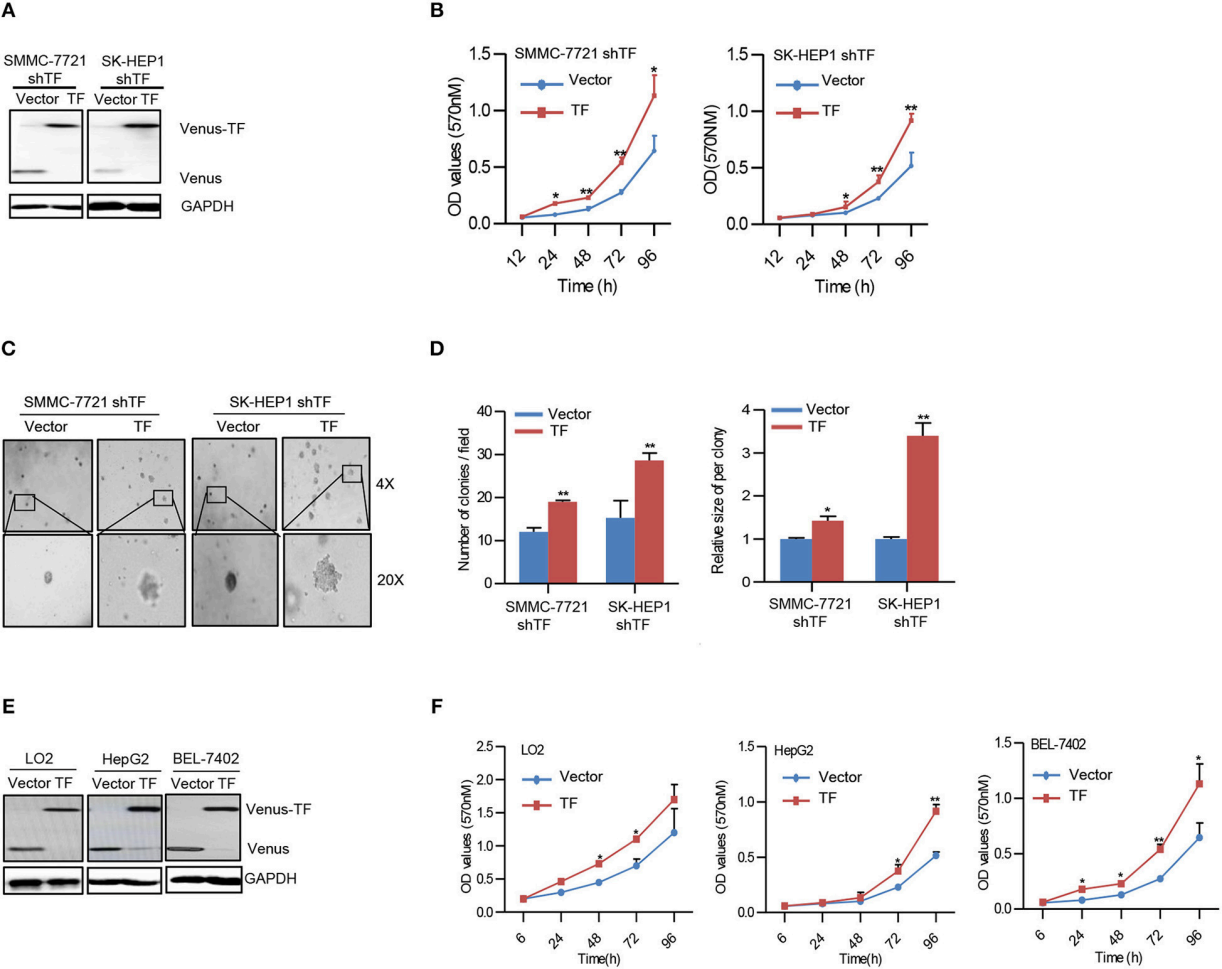
Overexpression of TF promotes the growth of HCC. **(A,E)** Western blot analysis of the protein expressions in the indicated cells. **(B,F)** Cell growth of the indicated cells as determined with MTT assay. **(C)** Representative images and **(D)** quantification of the indicated cells sphere as determined with sphere formation assay. Error bars, mean ± SD. *p < 0.05 and **p < 0.01 [two-tailed Student's *t*-test **(B**,**D**,**F)**].

To confirm the effect of TF on HCC growth, we performed rescue experiments by ectopic expression of TF in both TF-silenced SMMC-7721 and SK-HEP1 cells (Figure 2A). Ectopic expression of TF increased the cell amounts, sphere numbers, and sizes in both TF-silenced SMMC-7721 and SK-HEP1 cells (Figures 2B–D). Furthermore, overexpression of TF increased the cell amounts in LO2, HepG2, and BEL-7402 cells (Figures 2E, F). Taken together, these results suggest that TF can promote the growth of HCC.

### TF Promotes the Growth of HCC by Activating Both ERK and AKT Signaling Pathways

**Figure 3:**
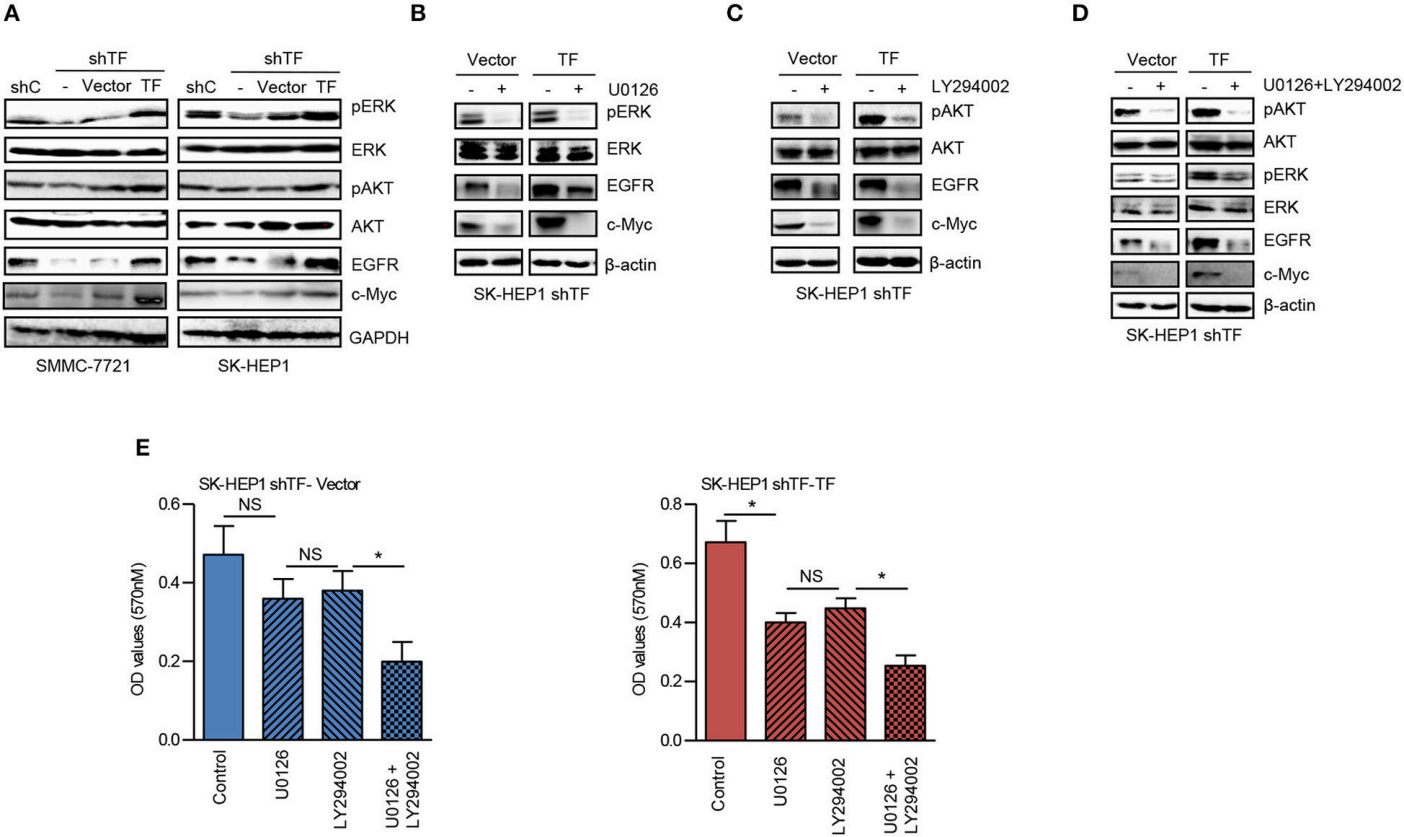
TF promotes the growth of HCC by activating both ERK and AKT signaling pathways. **(A)** Western blot analysis of the protein expressions in the indicated cells. SK-HEP1 shTF-Vector and SK-HEP1 shTF-TF cells were treated with/without U0126 and LY294002 at the concentration of 10 μM for 24 h. **(B–D)** Western blot and **(E)** MTT assay analysis of the protein expressions and cell growth. Error bars, mean ± SD. *p < 0.05 (two-tailed Student's *t*-test **E**).

To further explore the molecular mechanism of TF-promoted HCC growth, we detected the downstream signaling pathway of TF. As shown in Figure 3A, knockdown of TF decreased the protein levels of phosphorylated ERK (pERK), phosphorylated AKT (pAKT), and their downstream transcriptional factor c-Myc in both SMMC-7721 and SK-HEP1 cells. While ectopic expression of TF increased the protein levels of pERK, pAKT and c-Myc in both TF-silenced SMMC-7721 and SK-HEP1 cells. Interesting, the protein level of EGFR was downregulated in TF-silenced HCC cells and upregulated in TF-overexpressed HCC cells (Figure 3A). To define the roles of ERK and AKT in TF-mediated HCC growth, we examined the effects of MEK inhibitor U0126 and PI3K inhibitor LY294002 on the growth of both SK-HEP1 shTF-Vector and -TF cells. Treatment with U0126 or/and LY294002 decreased the protein levels of EGFR, c-Myc, pERK or/and pAKT in both SK-HEP1 shTF-Vector and -TF cells (Figures 3B–D). However, with U0126 or LY294002 alone inhibited the growth only in SK-HEP1 shTF-TF cells but not in SK-HEP1 shTF-Vector cells. After treating with the combination of U0126 and LY294002 significantly inhibited the growth in both SK-HEP1 shTF-Vector and -TF cells (Figure 3E). In short, these data suggest that TF promotes the growth of HCC by activating both ERK and AKT signaling pathways.

### Inhibition of EGFR Suppresses TF-Mediated HCC Growth

**Figure 4:**
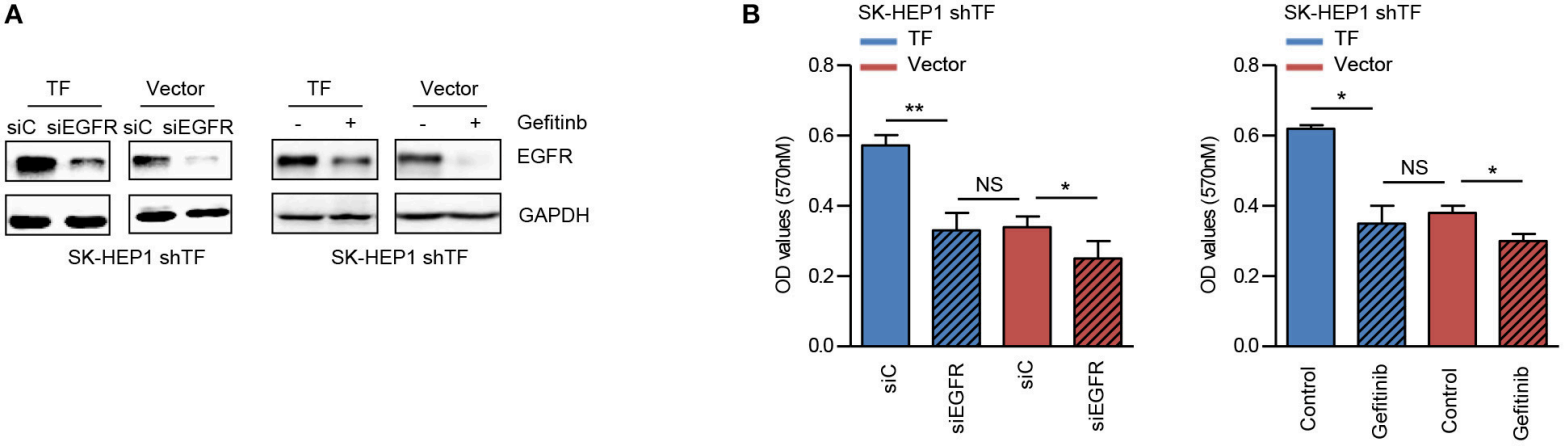
Inhibition of EGFR suppresses TF-mediated HCC growth. SK-HEP1 shTF-Vector and SK-HEP1 shTF-TF cells were transfected with siControl or siEGFR or treated with/without gefinib at the concentration of 10 μM for 24 h. **(A)** Western blot and **(B)** MTT assay analysis of the protein expressions and cell growth. Error bars, mean ± SD. *p < 0.05 and **p < 0.01 (two-tailed Student's *t*-test **B**).

EGFR has been identified as a key player in the development of HCC (25). To verify the role of EGFR in TF-mediated HCC growth, we examined the effects of EGFR siRNA and EGFR inhibitor gefitinib on the growth of both SK-HEP1 shTF-Vector and -TF cells. EGFR siRNA or gefitinib decreased the protein levels of EGFR in both SK-HEP1 shTF-Vector and -TF cells (Figure 4A). Furthermore, EGFR siRNA or gefitinib inhibited the growth more significantly in SK-HEP1 shTF-TF cells than in SK-HEP1 shTF-Vector cells, indicating that inhibition of EGFR suppresses TF-mediated HCC growth (Figure 4B).

### TF Protein Expression Is Correlated With EGFR in HCC Tissues

**Figure 5:**
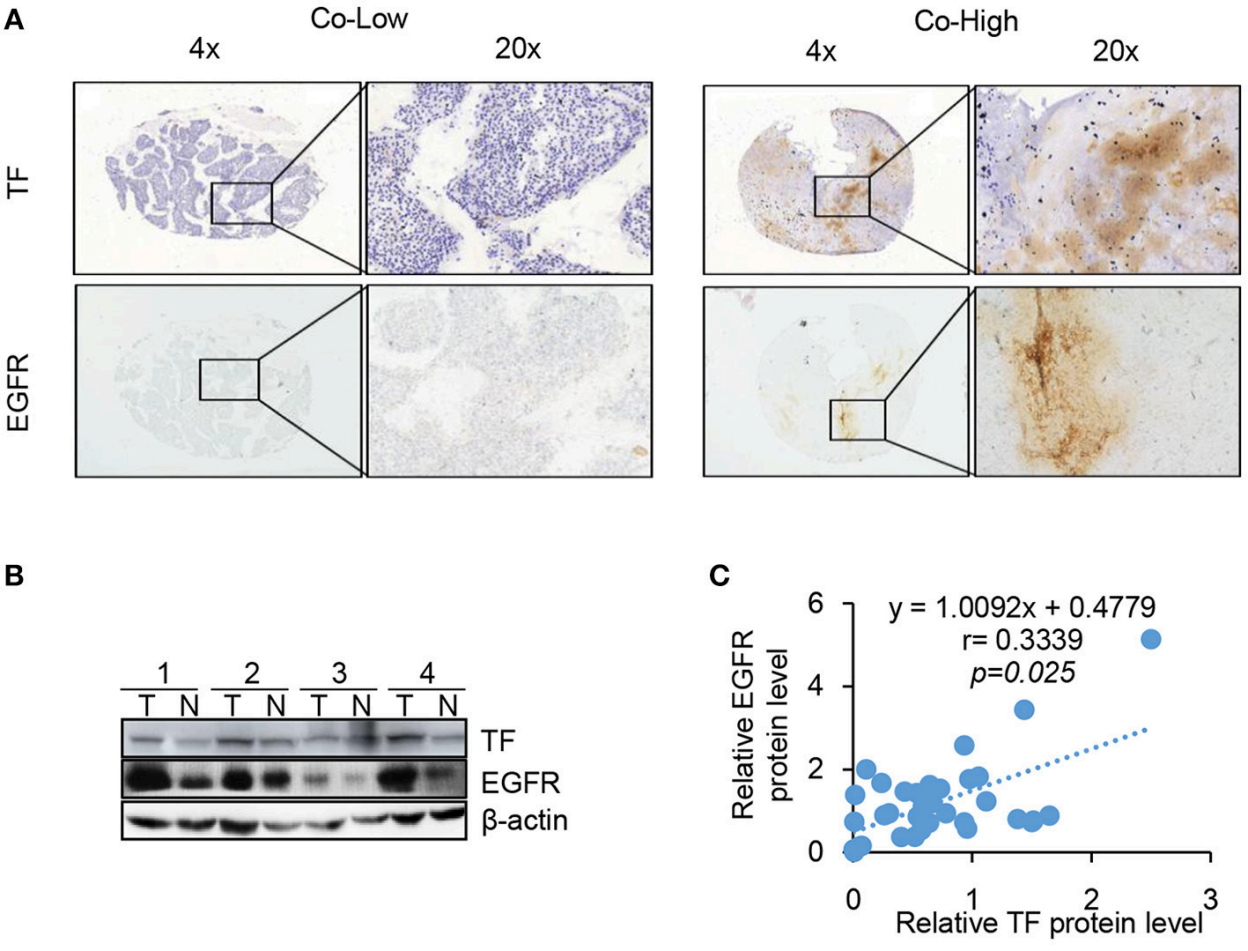
TF protein expression is correlated with EGFR and poor HCC patient prognosis. TF and EGFR protein expressions in 144 HCC tissues were examined with IHC assay. **(A)** Representative images of positive and negative expression of both TF and EGFR were shown at 4 X and 20 X magnification. **(B)** Representative images of western blot analysis of TF and EGFR protein expression in the paired HCC tissues and adjacent normal tissues. **(C)** Spearman's rank correlation test showed the correlation between TF and EGFR protein expressions by Western blot.

Our results clearly demonstrate that EGFR is regulated by TF in cell culture. To determine whether this is also the case in tumor tissues, we compared the protein levels of TF and EGFR in human 144 HCC tissues by IHC assay. High TF and EGFR staining were present in 105 (72.9%) and 91 (63.2%) out of 144 HCC tissues, respectively. Results of representative tissues with co-low or co-high staining of TF and EGFR were shown in Figure 5A. The expression of TF was highly correlated with the expression of EGFR in HCC tissues (Table 1 and Figures 5B, C).

**Table 1:** The correlation between TF and EGFR protein expressions in HCC tissues.

		**TF expression**			***P***
		**High**	**Low**	**Total**	**r**
EGFR	High	82	9	91	< 0.001
expression	Low	23	30	53	0.668
		105	39	144	

## Discussion

It has been demonstrated that TF-induced tumor progression need the activation of intracellular signaling pathways, where TF cytoplasmic domain couples to proteolytic activation of the protease activated receptor (PAR) 2 and subsequently activates ERK, AKT and other signaling pathways (26). For example, TF was involved in retinoblastoma cell proliferation via activating both ERK and AKT signaling pathways (27). Knockdown of TF suppressed human lung adenocarcinoma growth *in vitro* and *in vivo* through inhibiting both ERK and AKT signaling pathways (28). Similarly, our results showed that TF promoted the growth of HCC *in vitro* and *in vivo* by activating both ERK and AKT signaling pathways. Inhibition of ERK and AKT blocked TF-mediated growth of HCC. Therefore, activation of both ERK and AKT signaling pathways is indispensable for TF-promoted the growth of HCC.

EGFR is a member of ErbB/HER family of transmebrane receptor tyrosine kinases. It is activated by specific ligands resulting in the activation of multiple intracellular signaling pathways including ERK, AKT. Those signaling pathways is related to cell proliferation, migration and invasion (29–31). The gene expression of EGFR is regulated by the transcription factor c-Myc (32). In this study, we found that TF could enhance the expression of c-Myc and EGFR, and inhibition of ERK and AKT could block TF-induced c-Myc and EGFR upregulation. Phosphorylation of serine 62 amino acid residues by ERK prevents c-Myc protein from degradation (33). AKT stabilizes c-Myc protein by phosphorylation and inactivation of GSK-3β which phosphorylated threonine 58 amino acid residues of c-Myc to promote c-Myc degradation (33).

Inhibition of EGFR with either small molecule inhibitors or specific antibodies has achieved promising results in the preclinical HCC models. In human HCC cells, gefitinib, erlotinib or cetuximab could induce growth inhibition, cell cycle arrest and apoptosis (34–36). In the orthotopic HCC models, gefitinib significantly inhibited the growth and metastasis of HCC tumors, and enhanced by the combination with cisplatin (37, 38). However, the outcome of targeting EGFR in HCC was modest in the clinical trials. When used as a single agent in HCC patients, erlotinib only acquired moderate effects (39, 40), and cetuximab showed no antitumor activity (41). Treatment failure with EGFR inhibitors in HCC patients may cause by many reasons, such as the levels and mutations of EGFR, EMT status of tumor cells, etc. (42–44). In the current study, we found that treatment with EGFR siRNA or gefitinib suppressed the growth more significantly in the TF highly expressed HCC cells, suggesting that the levels of TF in tumor cells may influence the effects of EGFR inhibitors. Furthermore, our IHC data showed that both positive ratios of TF and EGFR protein in the HCC tissue were 72.9% (105/144) and 63.2% (91/144), respectively. The expression of TF was highly correlated with the expression of EGFR in HCC tissues. Therefore, it may be valuable to investigate the relation of TF expressions and EGFR inhibitors effects in the future studies.

## Conclusions

Our results provide proof-of-principle insights into a novel mechanism driven by TF on HCC growth and suggest that TF and EGFR may be potential therapeutic targets of HCC.

## Data Availability

The datasets generated for this study are available on request to the corresponding author.

## Author Contributions

S-ZH, M-NW, J-RH, Z-JZ, W-JZ, Q-WJ, and YY performed experiments. H-YW, H-LJ, KW, Z-HX, M-LY, and YL collected and analyzed data. X-SH, ZS, and QZ prepared the manuscript.

### Conflict of Interest Statement

The authors declare that the research was conducted in the absence of any commercial or financial relationships that could be construed as a potential conflict of interest.
